# Distributed Joint Source-Channel Coding in Wireless Sensor Networks

**DOI:** 10.3390/s90604901

**Published:** 2009-06-22

**Authors:** Xuqi Zhu, Yu Liu, Lin Zhang

**Affiliations:** School of Information and Communication Engineering, Beijing University of Posts and Telecommunications, 100876, Beijing, China; E-Mails: safiml@gmail.com (X.Z.), liuy@bupt.edu.cn (Y.L.)

**Keywords:** distributed joint source-channel coding, wireless sensor networks, multiple access channels, broadcast channels, information theory

## Abstract

Considering the fact that sensors are energy-limited and the wireless channel conditions in wireless sensor networks, there is an urgent need for a low-complexity coding method with high compression ratio and noise-resisted features. This paper reviews the progress made in distributed joint source-channel coding which can address this issue. The main existing deployments, from the theory to practice, of distributed joint source-channel coding over the independent channels, the multiple access channels and the broadcast channels are introduced, respectively. To this end, we also present a practical scheme for compressing multiple correlated sources over the independent channels. The simulation results demonstrate the desired efficiency.

## 1. Introduction

In recent years, the growing concern for our environment and society has led to applications ranging from detecting chemical leaks to monitoring underground parking. Wireless Sensor Networks (WSNs) have received significant attention to fulfill these requirements. WSNs consist of a large number of cheap sensors, e.g. intelligent sensor nodes, micro-cameras, which are densely allocated and self-organized to efficiently and reliably perform complex tasks in inaccessible situations.

While having a significant impact throughout society with their rich application space, WSNs also bring challenges to information and network technologies. The constrained energy of the sensors leads to limited processing capabilities and transmitting power. Furthermore, the impacts of noise, fading and multi-user channels [1] damage the data reconstruction. Therefore, for sending the collected data from sensors to the sink (a more powerful sensor node for preliminary data collection and processing), a robust method with high compression and low complexity is urgently needed in WSNs.

Addressing the above issues, Distributed Joint Source-Channel Coding (DJSCC) has attracted considerable research as an accepted efficient technique. DJSCC derives from the Distributed Source Coding (DSC), which is an important source coding development. DSC allows the separate encoding of correlated sources to be as efficient as joint encoding in traditional Shannon's theory, which is stated by the Slepian-Wolf theorem [2]. This advantage suits the WSNs perfectly by employing the large redundancy among the sensors' data collections and saving the energy for inter-sensor communications. Moreover, the joint source-channel approach is also an inevitable choice in WSNs. Since the separate design requires two sets of encoders and decoders for individual source coding and channel coding respectively, this aggravates the burden of the sensors in WSNs. Another reason is that the joint approach is usually more efficient or at least equal to the separate approach. Consequently, unifying the DSC and joint source-channel coding, DJSCC is fully efficient in WSNs.

This paper summarizes the developments of DJSCC over various channels in WSNs. In [3], the authors simply generalize three categories of situations: the asymmetric case, the independent channels case and the Multiple Access Channel (MAC) case. Usually we divide the sensors in WSNs into several clusters in which there is a more powerful sensor node acting as a sink for the preliminary collection and analysis of the data from the member sensors. When only one source is compressed and transmitted to the sink at a time, the sink uses its full information as the side information for jointly decoding which is known as the asymmetric case. If there is more than one source to be compressed, they can be transmitted through several independent channels or an MAC to the sink. However, reference [3] ignores another important case, the Broadcast Channel (BC) case, which has attracted attention in recent years. We add the fourth case because this will produce an adaptive WSN with minimum cost. The member sensors can receive a global observation from the sink in addition to their own measurements to make reliable decisions or other reactions. For example, the global observation can be a threshold of the interested temperature which may changes as required. The sink will distribute this global information to all the member sensors in time so that the member sensors can avoid transmitting useless data to the sink and save energy.

This paper is meant to generalize a framework for the development of DJSCC in WSNs, and is by no means exhaustive. The organization is as follows. A brief review of DSC is presented in Section 2, especially the noise immunity of the DSC. The previous theoretical results and implementing methods of the DJSCC for the asymmetric case, the independent channels case, the MAC case and the BC case are presented in Section 3. Our proposed scheme is provided as an example showing the efficiency of DJSCC for the independent channels case in Section 4. Section 5 concludes the paper.

## 2. Distributed Source Coding

### 2.1. Theory and Practice

Slepian-Wolf theorem [2] proves the achievable region of DSC for correlated sources *X*_1_ and *X*_2_:

(1) $$\begin{array}{c} R_{1}\geq H\left(X_{1}|X_{2}\right)\\ R_{2}\geq H\left(X_{2}|X_{1}\right)\\ R_{1}+R_{2}\geq H\left(X_{1},X_{2}\right) \end{array}$$

where *H(▪)*represents the entropy function, and *R_i_* is the encoding rate of *X_i_, i* = 1,2, It is called as the Slepian-Wolf rate region. In [4] the technique of random binning to generalize the Slepian–Wolf result to jointly ergodic sources was introduced. Similar results are obtained by [5] with regard to lossy coding. In brief, binning refers to partitioning the space of source into disjoint subsets or bins under conscious design. The goal is to reduce the bits used for identifying a source symbol with the index of the bins.

Reference [6] first introduced the practical strategy to exploit the potential of the Slepian-Wolf theorem based on channel codes. The statistical dependence between the two sources can be modeled as a virtual correlation channel. The source *X*_1_ and the other source *X*_2_ (called the side information) can be regarded as the input and the output of the virtual channel respectively. Thus, the DSC can be processed as the channel coding problem. Based on this idea, the first practical DSC scheme called DISCUS [7] was designed. So far there has been a flurry of practical DSC schemes [8-14].

### 2.2. Syndrome-Based and Parity-Based DSC

The binning/syndrome approach and the parity approach are major methods for practical DSC due to the notion in [6]. References [7,9,12,14] use the syndrome approach, while [8,10,11,13] use the parity approach. As analyzed in [15], the syndrome approach outperforms the parity approach by optimizing the space partitioning in the noiseless environment. However, in noisy environment, the syndrome approach is sensitive to any mistake in the syndrome sequence (bin-index), so the search space will shift to unforeseeable one. In comparison, the parity approach is much more robust to residual transmission errors. Moreover, the parity approach can directly use the encoders and decoders from channel codes. All the considerations above lead to the conclusion that parity-based DSC is more efficient in distributed compression with noisy communication channels, namely the DJSCC problem.

## 3. Distributed Joint Source-Channel Coding

### 3.1. DJSCC for the Asymmetric Case

**Figure 1.**
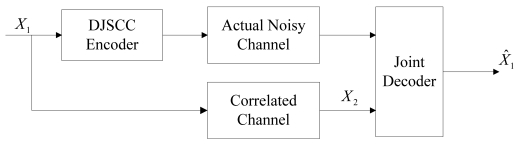
The system model of DJSCC for the asymmetric case.

In the asymmetric case, one of the sources *X_2_* is available at the decoder, and it can be treated as the noisy version of the source *X_1_* through a virtual correlated channel. The goal is to reconstruct *X_1_* lossless or within certain distortion at the joint decoder with the help of the side information *X_2_*. The system model is shown in Figure 1.

In the asymmetric case, the separation theorem is valid. [16] proved that the standard separation theorem [1] can be used while replacing the entropy of source by the conditional entropy with side information. Thus, the source *X_1_* can be reliably transmitted if and only if:

(2) $$H\left(X_{1}|X_{2}\right)<C$$

where *C* is the capacity of the actual noisy channel. Combined with the Slepian-Wolf theory *R*_1_ ≥ *H* (*X*_1_ | *X*_2_) the encoding rate of *X_1_* for the DJSCC in the asymmetric case can be derived that:

(3) $$R_{1}\geq H\left(X_{1}|X_{2}\right)/C$$

An extension to lossy DJSCC in this case is given in [17]. Assuming the sources discussed are independent, identically distributed binary sequences, the code is less efficient on compression than the one in a pure Slepian-Wolf scheme, for C < 1. However, we acquire the abilities of resisting the errors of the noisy channels. Therefore, the most direct method for implementing DJSCC is sending additional bits for general DSC scheme to approach the theoretical bound. Based on this idea, efficient designs are proposed in [18-23].

Reference [18] presents the case that decompression must be done from compressed data corrupted by Additive White Gaussian Noise (AWGN). Turbo codes are used by finely designing the matrices of the two constituent encoders. The design of [19] exploits systematic Irregular Repeat-Accumulate (IRA) codes [24] for DJSCC. The feasibility of designing different channel conditions for the systematic and the parity part separately is the main advantage. The simulation results confirm the superior performance to the turbo codes scheme. Using the IRA codes for precoding, a Raptor code [25] was designed for DJSCC [20] over packet erasure channels. The rateless property can guarantee the success in decoding regardless of the packet loss ratio. A bias towards selecting IRA parity symbols versus systematic symbols in forming the bipartite graph of the LT code is introduced. However, they didn't give a calculable method to determine the optimal design. In [21], the authors propose a scheme of compressing the sources with the memory correlation following a Hidden Markov Model (HMM). Requiring no previous knowledge of the HMM parameters at the encoder or decoder, the proposed scheme can perform well.

The syndrome-based approach can still be used in DJSCC by treating the syndromes as a special type of parity bits. Reference [22] designs an error resilient syndrome-based DJSCC scheme using convolutional codes based on the Asymmetric Syndrome-former Inverse-syndrome-former Framework (ASIF) proposed in [28]. Inspired by reference [22], reference [23] considers the syndrome-based LDPC decoding in the asymmetric DJSCC problem.

### 3.2. DJSCC for the Independent Channels Case

**Figure 2.**
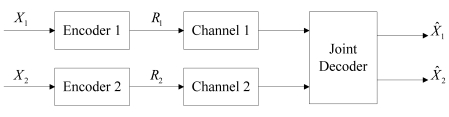
The system model of DJSCC for the independent channels case.

Instead of compressing only one source, in the independent channels case, all the sources are independently encoded and transmitted over their respective noisy channels, as shown in Figure 2.

**Figure 3.**
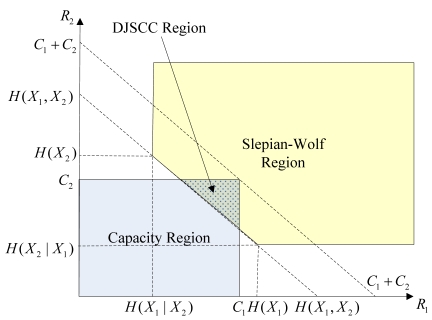
The DJSCC rate region for reliable transmission in the independent channels case: the intersection between the capacity region and the Slepian-Wolf rate region.

In the independent channels case, the separation theorem still holds [29]. It has been shown in [30] that an intersection between the channel capacity region and the Slepian-Wolf region is the sufficient and necessary condition for reliable communication, known as the independent channels theory. As illustrated in Figure 3, the conditions for two correlated sources *X_1_* and *X_2_* can be formulated as:

(4) $$\begin{array}{c} H\left(X_{1}|X_{2}\right)<C_{1}\\ H\left(X_{2}|X_{1}\right)<C_{2}\\ H\left(X_{1},X_{2}\right)<C_{1}+C_{2} \end{array}$$

where *C_i_* is the channel capacity of *X_i_, i* = *1,2*. Assuming *C_1_* = *C_2_* = *C*, the similar results with Formula (3) can be obtained as:

(5) $$\begin{array}{c} R_{1}\geq H\left(X_{1}|X_{2}\right)/C\\ R_{2}\geq H\left(X_{2}|X_{1}\right)/C\\ R_{1}+R_{2}\geq H\left(X_{1},X_{2}\right)/C \end{array}$$

where *R_i_* is the encoding rate of *X_i_*.

Typical designs [31-34] of DJSCC for the independent channels case follow the same strategy of that for the asymmetric case. In [31], the Low-Density Generator Matrices (LDGM codes) [35] are used for DJSCC. A concatenated scheme is proposed for avoiding error floors. Combining the sparseness of the generator matrix and the parity check matrix, LDGM codes present a complexity advantage over standard LDPC and turbo codes. The performance of the proposed DJSCC scheme is very close to the theoretical limits in practical situations. A DJSCC scheme for known/unknown correlated sources using “super” turbo code is proposed in [32]. The so-called “super” turbo code consists of the turbo code for each source and an additional spread interleaver acting over the sequence associated with the second source. If the parameter of the correlation is unknown at the decoder, it still can be estimated in the decoding iterations. Extracting the similarity of [31] and [32], turbo-like codes for DJSCC is discussed in [33]. In [34], the authors consider the DJSCC problem using LDPC codes in a situation that no side information is communicated to the decoder. They also provide analytical performance bounds of joint iterative LDPC decoding of correlated sources.

### 3.3. DJSCC for the Multiple Access Channel Case

Most of the works on DJSCC over MAC concentrate on the necessary and sufficient conditions for sending the correlated sources with arbitrarily small probability of error. The practical algorithms are rare to the best of our knowledge.

**Figure 4.**
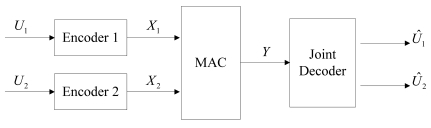
The system model of DJSCC for the MAC case.

Reference [36] provides the theorem developing the so-called single letter characterizations of an achievable rate region for correlated sources sent over a MAC. The system model is shown in Figure 4. Considering discrete alphabet sources (*U*_1_,*U*_2_) ∼ *p*(*u*_1_,*u*_2_), the sources transmit their codewords *X_i_, i* = {1,2}, to a single decoder through a memoryless MAC (*X*_1_ × *X*_2_, *Y, p* (*y*∣*x*_1_,*x*_2_)). The achievable region sending *U_1_* and *U_2_* with arbitrarily small error is:

(6) $$\begin{array}{c} H\left(U_{1}|U_{2}\right)<I\left(X_{1};Y|X_{2},U_{2}\right)\\ H\left(U_{2}|U_{1}\right)<I\left(X_{2};Y|X_{1},U_{1}\right)\\ H\left(U_{1},U_{2}\right)<I\left(X_{1},X_{2};Y\right) \end{array}$$

for some *p*(*u*_1_,*u*_2_,*x*_1_,*x*_2_,*y*) = *p*(*u*_1_,*u*_2_)*p*(*x*_1_∣*u*_1_) *p*(*x*_2_∣*u*_2_) *p*(*y*∣*x*_1_,*x*_2_). The MAC region and the Slepian-Wolf region are the special cases of the region in (6). Generalizing the results to sources with a common part *W* = *f*(*U*_1_) = *g*(*U*_2_), corresponding achievable rate region is listed in Theorem 1 of [36].

Although an instructive counter example is presented in [37] showing that the coding strategy of [36] is not optimal, the problem of determining the true capacity regions is open. Thus, many follow-up works are still based on the conditions of [36]. A graph-based modular framework is proposed in [38] to further minimize the performance gap. Taking the schemes in [36] as the elements, correlated sources transmission over MACs with receiver side information *Z* is discussed in [39]. The conditions for lossless representation are:

(7) $$\begin{array}{c} H\left(U_{1}|U_{2},Z\right)<I\left(X_{1};Y|X_{2},U_{2},Z\right)\\ H\left(U_{2}|U_{1},Z\right)<I\left(X_{2};Y|X_{1},U_{1},Z\right)\\ H\left(U_{1},U_{2}|Z\right)<I\left(X_{1},X_{2};Y|Z\right) \end{array}$$

for some joint distribution *p*(*u*_1_,*u*_2_,*z,x*_1_,*x*_2_,*y*) = *p*(*u*_1_,*u*_2_,*z*)*p*(*x*_1_∣*u*_1_) *p*(*x*_2_∣*u*_2_) *p*(*y*∣*x*_1_,*x*_2_). Note that the correlation among the sources condenses the region. Recently, [40] proposed a generalized theorem including the side information scenario and various channel conditions. The most important conclusion in [40] is that in the MAC case the joint designs outperform the Shannon separation limit.

Using the similar strategies in [31], a DJSCC scheme with LDGM over MAC is proposed in [41] and [42]. By utilizing different structures at the encoder site, the proposed system is able to outperform the separation limit for a wide correlation range.

Here one important point should be added, that there is a special case of MAC, discrete memoryless (dm) Asymmetric MAC (AMAC), over which the separation theorem can be applied [43]. In the dm AMAC situation, the messages of one source are encoded by both encoders, whereas the messages of another source are encoded by only one of the encoders. [43] derives the necessary and sufficient conditions for the reliable transmission in dm AMAC. The similar conclusions are presented in [44] considering the common source of the two sources is transmitted losslessly in the Shannon sense.

### 3.4. DJSCC for the Broadcast Channel Case

**Figure 5.**
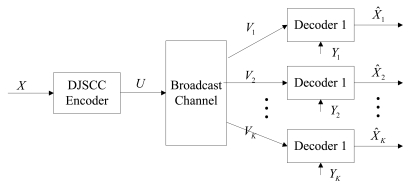
The system model of DJSCC for the BC case.

The problem of transmitting correlated sources over BC has been investigated as early as in 1987 [45]. However, the scenario they mentioned is not what we discuss here because the correlated sources are all available at the encoder. Many other results are proposed in the same settings [46,47]. We consider another situation that the side information is only known at the decoder. As shown in Figure 5, a memoryless source *X* is transmitted over a memoryless BC *P_V_*_1_,…,*_VK_*_|_*_U_*(*v*_1_,…,*v_K_*|*u*), where each receiver's own observation *Y_i_, i* = 1,…, *K* acts as the side information about the source *X*.

In [48], the author discusses the characterization of achievable rates for lossless coding using separate and joint designs. By analyzing the performance of both, joint source-channel coding allows a much simpler strategy for any K ≥ 2 and perfect single-letter nature. The rate of each channel per symbol *κ* is an achievable rate if and only if there exists *P_U_*(*u*) such that:

(8) $$H\left(X|Y_{i}\right)<\kappa I\left(U;V_{i}\right)$$

Then the necessary and sufficient condition for joint source-channel coding over BC is presented in Theorem 6 in [48] which states that reliable communication is possible with rate *κ* if and only if :

(9) $$\left(H\left(X|Y_{1}\right),H\left(X|Y_{2}\right),\ldots ,H\left(X|Y_{K}\right)\right)\in \kappa ℜ$$

is satisfied, where R is a tight bound on the total rates. The proof employs “virtual binning” and “operational separation” exhibited by the optimal joint source-channel coding strategy.

Using ideas from [48] and dirty paper coding [49], a design for lossy coding is proposed in [50]. The most direct extension is adding quantization before transmission, whose sufficient condition is given in Theorem 1 of [50]. A dirty-paper version of Theorem 1 is proposed by additionally handling channel state information (CSI) at the encoder in Theorem 2 in [50]. By numerical comparisons, their distortion performance is analyzed for the quadratic Gaussian and binary Hamming cases. Both of the schemes they designed are always at least as good as (in fact, except for one certain case, always better than) separate coding. In the subsequent paper [51], they combine the two digital schemes in [50] with analog or uncoded transmission to extract the benefits of both methods.

A brief summary of this section is given here. The first two cases, the asymmetric case and the independent channels case, are comparatively simple, and have been adequately studied. However, in the MAC case and the BC case, a number of problems remain to be solved; for example, the optimal rate regions of the general DJSCC settings and corresponding code design methods are all undetermined. If the interference of the adjacent channels can be considered as a kind of noise in WSNs, the MAC case degrades to the independent channels case. The research on the independent channels case will play a fundamental role in the study on DJSCC. Thus, we propose a DJSCC scheme for multiple correlated sources over the independent channels in the following section.

## 4. Proposed Scheme of DJSCC for the Independent Channels Case

A practical DJSCC scheme for multiple correlated sources is proposed in the scenario of the independent channels case. Compared with the practical designs listed in subsection 3.2, the proposed scheme stresses two points. Firstly, the DJSCC for three correlated sources is investigated. Intuitively, this leads to more efficient redundancy utilization than considering pairs. In contrast, the approaches in [31-34] all process two correlated sources. Though [52-54] have considered the compression of three correlated sources, the actual noisy channels are all absent in them. Secondly, the noisy side information is considered in the theoretical limits determination and the optimal code design; moreover, an instructive extension of the previous independent channels theory [29-30] is derived.

In the proposed scheme, an efficient coding strategy with a single LDPC code is designed to cope with both source and channel coding. And the theoretical limits of DJSCC for multiple sources are derived. The simulation results demonstrate the desired efficiency of the proposed DJSCC scheme.

### 4.1. Proposed Scheme

**Figure 6.**
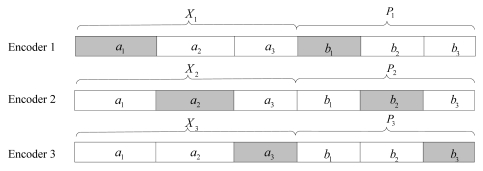
Structures of the outputs of the encoders: Only the gray squares are transmitted for each encoder.

The proposed scheme is inspired from the parallel channel model introduced in [54]. The basic idea is dividing the encoded codeword into several fractions in order to process them separately. Consider three memoryless binary sources *X_1_, X_2_* and *X_3_* which are statistically dependent to each other with cross-over probability P[*X_j_* ≠ *X_i_* ∣ *X_i_* ]= P_ij_, ∀*i, j* ∈ {1,2,3} and *i* ≠ *j*. The *k* - bit sequence of source *X_i_* is encoded independently using a common systematic LDPC code *(k,n)*; thus, the codeword of *X_i_* consists of the information bits *X_i_* and the parity bits *Pi*, where ∣*P_i_*∣ = *n - k*. However, it is unnecessary to transmit all the codewords to the decoder for the existence of correlation. So for source *X_i_*, only the *a_i_k* information bits and *b_i_*∣*P_i_*∣ parity bits are transmitted through the noisy channel, where *a_i_* and *b_i_* are the proportional coefficients, 0 ≤ *a_i_,b_i_*≤ 1, $\sum_{i=1}^{3}a_{i}=\sum_{i=1}^{3}b_{i}=1$. The structures for transmission are illustrated in Figure 6. The DJSCC encoding rate *R_i_* for *X_i_* is calculated as:

(10) $$R_{i}=\frac{a_{i}k+b_{i}|P_{i}|}{k}$$

Since *R_i_* has the constraint condition according to the independent channels theory, the selection of *a_i_* and *b_i_* also follow the rules which will be discussed in the next subsection.

At the joint decoder, the received different parts of the information bits from different encoders will re-produce an integrated codeword for decoding, and they act as side information for each other. Therefore, the initial LLRs for different fractions in message-passing algorithm for decoding [9] are different according to different channel models.

### 4.2. Correlation Model and Theoretical Limits

Suppose that the actual noisy channel for the source *X_i_* is the AWGN channel with variance $\sigma_{i}^{2}$, assuming $\sigma_{i}^{2}=\sigma^{2}$ for clarity. Firstly considering *X_1_*, for obtaining a complete codeword for decoding, the corresponding *a*_2_ and *a*_3_ fractions are replaced by the side information which are received from *X_2_* and *X_3_* respectively. Since the side information is also corrupted by the AWGN, the correlation between the side information and the original information is modeled as a combined channel of the virtual BSC and the AWGN channel. This method can be applied in decoding for *X_2_* and *X_3_* similarly.

**Figure 7.**
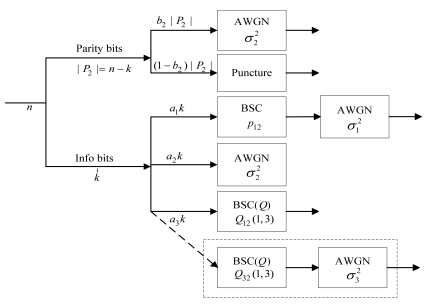
The parallel channel model of the source *X_2_* for three source DJSCC: The dashed box shows an alternative situation that we take *X_3_* rather than *X_1_* as the input of the virtual combined channel BSC (*Q*).

However, there is a more complex situation in determining the correlation models for some fractions of *X_2_* and *X_3_*. Taking *X_2_* as an example, there are two kinds of side information for the a_3_ fraction, the decoded version X̂_1_ of *X_1_* and the received version of *X_3_*. As introduced in [52], we can model this dependency by a BSC with an unfixed cross-over probability *Q*, denoted by BSC (*Q*). The value of *Q* is determined by whether the decoded version X̂_1_ and the received version of *X_3_* are equal. Figure 7 demonstrates the parallel channel model of source *X_2_*, which reveals the correlation model for different fractions of it. Other source can be modeled in the same manner.

The remainder of this subsection will give the constraint condition of *R_i_* for this model. Assuming *P_ij_* = *P*, the channel capacities of the noisy channel, the combined BSC+AWGN channel and the combined BSC(*Q*)+AWGN channel are represented by *C*(*σ*^2^), *C*(*p, σ^2^*) and *C*(*Q, σ^2^*) respectively.

**Theorem:** The encoding rates of *X_1_, X_2_, X_3_* and the total encoding rate follow the constraints:

(11) $$R_{1}\geq \frac{1-(1-a_{1})C(p,\sigma^{2})}{C(\sigma^{2})}$$

(12) $$R_{2}\geq \frac{1-a_{1}C(p,\sigma^{2})-a_{3}C(Q,\sigma^{2})}{C(\sigma^{2})}$$

(13) $$R_{3}\geq \frac{1-(1-a_{3})C(Q,\sigma^{2})}{C(\sigma^{2})}$$

(14) $$R_{1}+R_{2}+R_{3}\geq \frac{3-C(p,\sigma^{2})-C(Q,\sigma^{2})}{C(\sigma^{2})}$$

**Proof:** Taking source *X*_2_ as an example, the overall channel capacity of *X*_2_ is:

(15) $$C_{2}^{\text{sum}}=a_{2}R_{2}^{c}C(\sigma^{2})+(1-R_{2}^{c})C(\sigma^{2})+a_{1}R_{2}^{c}C(p,\sigma^{2})+a_{3}R_{2}^{c}C(Q,\sigma^{2})$$

where the code rate of this channel is $R_{2}^{c}$ and $R_{2}^{c}\leq C_{2}^{\text{sum}}$, resulting:

(16) $$R_{2}^{c}\leq \frac{C(\sigma^{2})}{1-a_{2}C(\sigma^{2})+C(\sigma^{2})-a_{1}C(p,\sigma^{2})-a_{3}C(Q,\sigma^{2})}$$

Since $R_{2}^{c}=k/\left(k+\left|P_{2}\right|\right)$, the numbers of the parity bits ∣*P*_2_∣have the lower bound:

(17) $$\left|P_{2}\right|\geq \frac{[1-a_{1}C(p,\sigma^{2})-a_{3}C(Q,\sigma^{2})]k}{C(\sigma^{2})}-a_{2}k$$

This implies the formula (12) by substituting (10) into the (17). Similarly, other results of this theorem can be achieved.

In order to compare with the previous theoretical results in subsection 3.2, we consider the case of two correlated sources *X_1_* and *X_2_*. This is a special case of our proposed scheme which can be obtained by setting *a*_1_ = *a, a*_2_ = 1 - *a, a*_3_ = 0, *b*_1_ = 1-*a, b*_2_ = *a* and *b*_3_ = 0, where 0≤a≤1. The total encoding rate can be obtained as:

(18) $$R_{1}+R_{2}\geq \frac{2-C(p,\sigma^{2})}{C(\sigma^{2})}$$

Neglecting the noisy influence on the side information bits, we can get C(p,σ^2^)=C(p)=1–H(p). Introducing it into formula (18), the same representation of formula (5) is achieved. Thus, the independent channels theory in subsection 3.2 is a special case of our theorem. This is a novel and instructive extension of the previous independent channels theory. Considering the noise interference on the side information bits, as the scheme we proposed, is more reasonable in practical applications.

### 4.3. Simulation Results and Analysis

In order to evaluate the performance of the proposed DJSCC scheme, we simulate it for the two sources and three sources cases. The length of original source sequence is fixed to *k* = 10^8^. The LDPC code we used is defined by the degree distribution (19) from [55]. Here *λ* (*x*) denotes the variable-node degree distribution of the LDPC code, and the check-node degree is approximately uniform.

(19) $$\lambda (x)=0.170031x+0.160460x^{2}+0.112837x^{5}+0.047489x^{6}+0.011481x^{9}+0.091537x^{10}+0.152978x^{25}+0.036131x^{26}+0.217056x^{99}$$

#### 4.3.1. Two Sources Case

**Figure 8.**
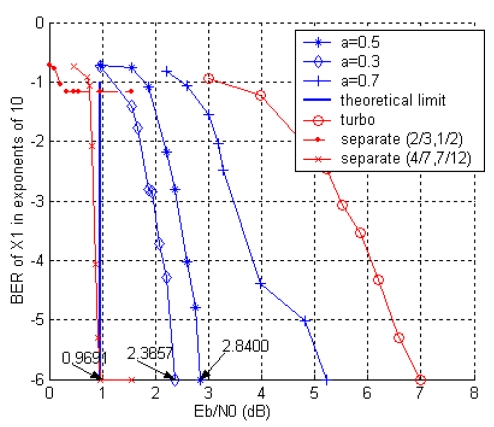
BER of the *X_1_* in the two sources DJSCC scheme.

It is assumed that the cross-over probability between the two sources *p* = 0.1. The code rate is *R^c^* = *k*/(*k* + ∣*P*∣) = 1/3, and the total encoding rate is *R* = *R*_1_ + *R*_2_ = 1/*R^c^* = 3. For the given *p* and *R*, the theoretical bound of the *E_b_*/*N_0_* can be calculated as 0.9691 dB for lossless recovery. We consider a symmetric rate case with *a* = 0.5 and two arbitrary rate cases with *a* = 0.3 and *a* = 0.7 respectively. The Figure 8 demonstrates that as *a* increases, the error correcting capabilities decreases. It can be found that our scheme is superior to the one proposed in [33] with turbo codes.

We also simulate two separate designs with the same total encoding rate *R* = 3, where the source code and channel code rate pairs (*R^s^, R^c^*) are (2/3, 1/2) and (4/7, 7/12). The convergence value is restricted by the source code when the channel code can completely remove the error, while in DJSCC scheme the surplus parity bits for channel code can help to correct the source errors. The separate design can be superior to DJSCC (see the curve of separate (4/7, 7/12) case), but complexity will significantly increase accordingly.

#### 4.3.2. Three Sources Case

**Figure 9.**
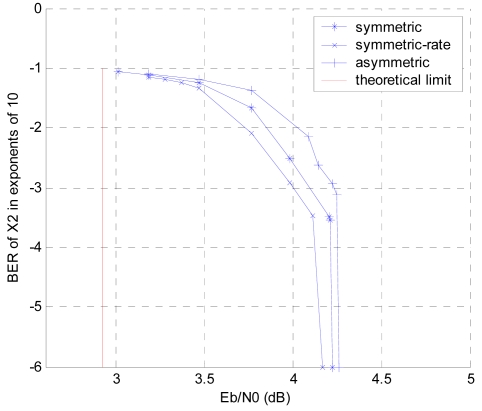
BER of the *X_2_* in the three sources DJSCC scheme.

**Figure 10.**
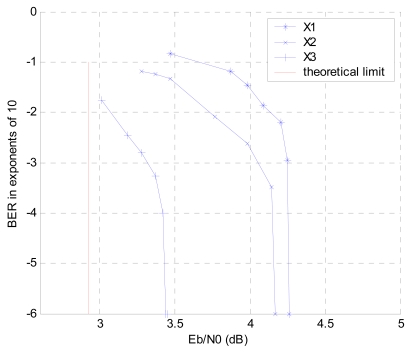
BER of the sources *X_1_, X_2_* and *X_3_* in the symmetric-rate case.

In our paradigm, it is assumed that *p_ij_* = *p* = 0.1, *R^c^* = 1/3. The theoretical bound here is 2.9243 dB. We first consider a symmetric setting with *a_i_*= *b_i_* = 1/3; then, a symmetric-rate setting (*R_i_* = 1 = *R*/3) with *a_1_* = 0.28, *a_2_* = 0.34, *a_3_* = 0.38, *b_1_* = 0.36, *b_2_* = 0.33 and *b_3_* = 0.31 is simulated. Finally, an asymmetric setting which satisfies the rate limits in formulas (11-14) is considered, in which we calculate *a_1_* = 0.28, *a_2_* = 0.34, *a_3_* = 0.38, *b_1_* = 0.373, *b_2_* = 0.315 and *b_3_* = 0.312 with the *E_b_/N_0_* value of the theoretical limit. As shown in Figure 9, the asymmetric case with the least parity bits converges slowest even though it has more information bits than other settings. With the same amount of parity bits, the symmetric-rate case performs better than the symmetric case for more information bits. The performance of BER for all the three sources *X_1_, X_2_* and *X_3_* in the symmetric-rate case is shown in Figure 10. It can be noted that, as expected, from *X_1_* to *X_3_* the convergence values are closer to the theoretical bound because more side information is used at decoding for the latter decoded source.

## 5. Conclusions and Future Work

In this paper, a survey of the theories and implementation approaches of DJSCC for the asymmetric case, the independent channels case, the multiple access channel case and the broadcast channel case is presented. We have proposed an efficient framework of DJSCC for the independent channels case. Our results extend the existed theoretical limits of DJSCC to a more practical scenario by considering the noisy side information and multiple correlated sources. The simulate results verify the limit-approaching performance of the proposed scheme.

It can be found that the problem of finding the necessary and sufficient conditions of the most general setting in the MAC case and the BC case remains open to this day. Moreover, corresponding efficient coding strategies also have a tremendous space for further development. One of the main issues for practical deployment of DJSCC is the correlation model. In practical WSNs, it is usually hard to determine the exact joint distributed function; moreover, the correlation may be time-varying. Thus, the adaptive DJSCC scheme is a meaningful and challenging problem. Another future direction is introducing the multiterminal source coding to DJSCC. Since the multiple access channels and the broadcast channels are all the multi-user channels, it is obvious that the distributed compression of multiple correlated sources should be considered. However, the existing designs for DJSCC mainly constrained on processing two sources. The proposed scheme we presented here represents a first step towards this goal.
